# Editorial: Institutional Determinants of Social Inequalities

**DOI:** 10.3389/fpsyg.2015.02027

**Published:** 2016-01-08

**Authors:** Frédérique Autin, Fabrizio Butera

**Affiliations:** UNILaPS, Faculté des Sciences Sociales et Politiques, Institut de Psychologie, Université de LausanneLausanne, Switzerland

**Keywords:** social inequalities, institutions, education, social reproduction

To understand the persistence of social inequalities, research in psychology has traditionally focused on individual determinants of the unequal treatment of social groups. For example, a great deal of work has situated the origin of inequalities in the discrimination produced by individuals who are biased by their negative beliefs and attitudes. And yet, several authors proposed that individuals' action and psychological tendencies are grounded in (and foster) the social world (e.g., Fiske et al., 1998). As a result, determinants of inequalities could be better analyzed by considering the way in which the social world is structured and shape people's experiences (see Adams et al., 2008). In particular, institutions (e.g., educational systems, politics and the legal system, media) are an important structural element that shapes people's experiences. Institutions reflect and promote ideas and values (e.g., equal opportunities, meritocracy, etc.), and thereby influence the way people think about themselves, others and society. However, some scholars proposed that institutions reflect the perspective of, and are structured to benefit the dominant groups (Jackman, 1994; Adams et al., 2008; Markus and Fiske, 2012). They convey ideas, promote norms, and legitimate practices that maintain and justify existing inequalities. Consequently, institutions participate in enhancing the experiences of dominant group members, while hindering those of dominated group members.

The present research topic in *Frontiers in Educational Psychology* proposes to bring together recent research studying how institutions favor dominant groups and disadvantage dominated groups. It gathers contributions from educational sciences, social psychology, cultural psychology, and sociology. Various forms of inequalities are investigated, based on social class, gender, nationality group, and migratory status. A broad range of institutional factors are considered as potential determinants of inequalities: national asylum policies and economic disparities, ideologies and practices embedded in educational institutions such as schools, universities and museums, recruitment, and human resources practices.

In an opinion piece, Sanchez-Mazas discusses the fundamental right of children to education, within the framework of migration. In particular, she analyses the asylum policies that might deny migrant such a fundamental right, with a focus on policies that trigger the disappearance of failed asylum seekers into clandestinity.

The next four articles report research that investigates how some peculiarities of educational institutions might contribute to inequalities. This research highlights barriers to the success of women and low social class students that are embedded in the very structure of educational institutions. Wiederkehr, Bonnot, Krauth-Gruber, and Darnon show that the widespread belief in meritocracy at school (i.e., success depends on hard work) serves a system-justifying role for low status students. This work points to the problem that schools might convey ideological beliefs that reinforce low status individuals' acceptance of their lower position in society. Jury, Smeding, and Darnon focus on the function of selection of educational institutions, that is their role in identifying the best students, rewarding them with degrees and guiding them toward the highest social positions. They demonstrate that merely reminding students of the selection operated at university hinders the performance of first-generation students. The function of selection would thus contribute to the achievement gap between first- and continuing-generation students. Autin, Batruch and Butera also studied the function of selection. Results showed that endorsing the idea that educational institutions should select the best student predicts more support for traditional assessment practices—although known to disadvantage low status students—and less support for alternative assessment practices. Promoting the idea that schools select the most deserving students would thus restrain changes in assessment practices toward greater equality. Finally, Sommet, Quiamzade, Jury, and Mugny propose to unravel institutional obstacles to the success of both low and high status students. Their results suggest that a competitive academic context reduces learning goal endorsement in first-generation students but that a less competitive context reduces learning goals in continuing-generation students. This work pleads for a greater consideration of the interaction between the students' status and the structure of the educational institutions.

Mukherjee, Salter, and Molina broaden the range of educational institutions and analyze museums as tools for history education. They propose that the historical representation of immigration reflects the dominant group's identity. Reciprocally, engaging in such representation of history shapes visitors' experience to favor the dominant group, for example by increasing exclusive stances toward immigrants.

The next two articles tackle the interplay between the educational and the professional world. Jensen and Jetten investigate student's professional and academic identity development. They question how the interactions organized in universities foster or, on the contrary, restrain the creation of the social capital that facilitates identity formation. Their results show that interactions with other students facilitates the emergence of academic identity, but hinders interaction with teachers and the emergence of a professional identity. Goastellec and Ruiz focus on those students at the crossroad between schools and companies: apprentices. These authors highlight how the criteria used to recruit apprentices, such as skills and knowledge not taught at school, tend to reproduce already existing social class inequalities.

The two final articles widen the scope of the research topic by studying institutional inequalities in the corporate world. Maitner and DeCoster show that economic inequalities between nations are transmitted to expected inequalities in payment of individuals from these different nations, thus reproducing the global hierarchy at an organizational level. Starmarski and Son Hing review the organizational determinants of gender inequalities in the workplace and propose a model of gender discrimination in human resources.

Institutions are pervasive and powerful structures in everyday life. The research gathered in this research topic questions the neutrality of these institutions in terms of power relations between social groups. The different research streams point out factors that contribute to social inequalities but are subtle and hard to identify as such because they are embedded in the institutions that shape people's experiences. The ideas and findings presented in this research topic offer several contributions to the growing literature on the institutional determinants of social inequalities.

## Author contributions

FA drafted the manuscript and FB provided critical revisions. All authors approved the final version of the manuscript for submission.

## Funding

This research was funded by a Sinergia grant of the Swiss National Science Foundation.

### Conflict of interest statement

The authors declare that the research was conducted in the absence of any commercial or financial relationships that could be construed as a potential conflict of interest.
